# Genetic Risk Factors for Ischemic and Hemorrhagic Stroke

**DOI:** 10.1007/s11886-016-0804-z

**Published:** 2016-10-31

**Authors:** Ganesh Chauhan, Stéphanie Debette

**Affiliations:** 1 Inserm U1219 Bordeaux Population Health Research Center, 146, rue Léo Saignat, 33000 Bordeaux, France; 2 University of Bordeaux, Bordeaux, France; 3 Centre for Brain Research, Indian Institute of Science, Bangalore, India; 4 Department of Neurology, Bordeaux University Hospital, Bordeaux, France

**Keywords:** Stroke, Ischemic stroke, Hemorrhagic stroke, Genome-wide association studies, Multifactorial

## Abstract

Understanding the genetic risk factors for stroke is an essential step to decipher the underlying mechanisms, facilitate the identification of novel therapeutic targets, and optimize the design of prevention strategies. A very small proportion of strokes are attributable to monogenic conditions, the vast majority being multifactorial, with multiple genetic and environmental risk factors of small effect size. Genome-wide association studies and large international consortia have been instrumental in finding genetic risk factors for stroke. While initial studies identified risk loci for specific stroke subtypes, more recent studies also revealed loci associated with all stroke and all ischemic stroke. Risk loci for ischemic stroke and its subtypes have been implicated in atrial fibrillation (*PITX2* and *ZFHX3*), coronary artery disease (*ABO*, *chr9p21*, *HDAC9*, and *ALDH2*), blood pressure (*ALDH2* and *HDAC9*), pericyte and smooth muscle cell development (*FOXF2*), coagulation (*HABP2*), carotid plaque formation (*MMP12*), and neuro-inflammation (*TSPAN2*). For hemorrhagic stroke, two loci (*APOE* and *PMF1*) have been identified.

## Introduction

**Fig. 1:**
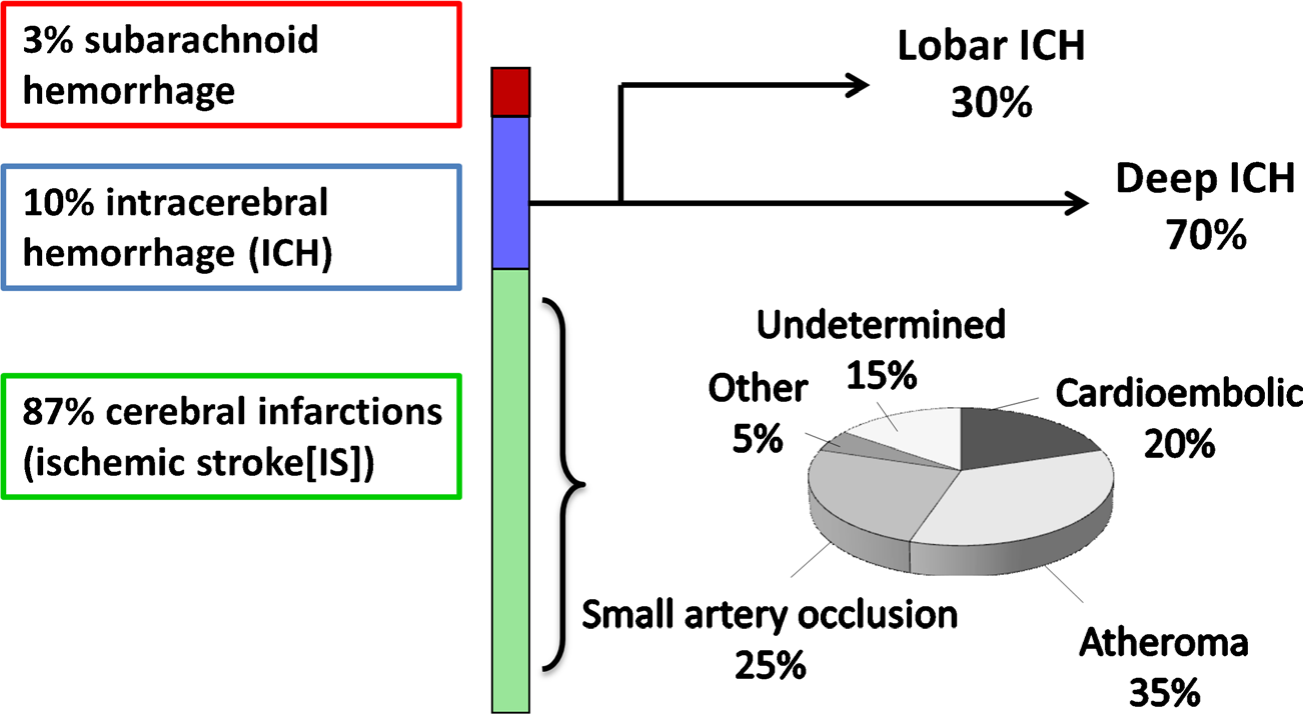
Heterogeneity of the stroke phenotype. Numbers are taken from the latest statement of the American Heart Association/American Stroke Association [116]

Stroke, characterized by a neurological deficit of sudden onset, typically due to brain infarction (“ischemic stroke”) or, less often, intracerebral hemorrhage, represents the primary neurological cause of acquired disability in adults and a leading cause of death [1]. It is also a major contributor to cognitive decline and dementia [2–4]. Common causes (subtypes) of ischemic stroke include large artery atheroma and cardiac sources of embolism, while small artery disease is a major cause of both ischemic stroke and intracerebral hemorrhage (Fig. 1) [5]. The lifetime risk of stroke has been estimated at one in five for middle-aged women and one in six for middle-aged men in the Framingham Heart Study [6]. A substantial proportion of stroke risk remains unexplained, and a contribution of genetic factors is supported by recent discoveries of common genetic variation associated with stroke risk, identified through large, collaborative, genome-wide association studies (GWAS) [7]. In rare instances, stroke can be directly caused by monogenic disorders, i.e., a rare mutation in one gene is sufficient to cause the disease. In the vast majority of cases, however, genetic risk factors contribute to the risk of stroke as part of a multifactorial predisposition, where each genetic variation is responsible only for modest increases in risk. The advent of high throughput genotyping in the past decade has led to progress in the discovery of genes underlying complex forms of stroke [7]. An important challenge in the identification of genetic determinants of stroke, in contrast with other common vascular or neurological diseases, is the complexity of the phenotype. Indeed, stroke is a highly heterogeneous condition that can be caused by multiple, extremely diverse etiologies (Fig. 1). Of note, genetic risk factors for subarachnoid hemorrhage will not be discussed in this review, as the underlying mechanisms are completely distinct from those of intracerebral hemorrhage and ischemic stroke.

The main application expected from these discoveries is to improve our understanding of the biological pathways underlying the disease, and, through this, to accelerate the identification of novel drug targets [8]. While improved risk prediction also remains a long-term goal, its implementation is still complex given the small effect size of genetic risk variants. With the expected increase of identified stroke risk variants through large international consortia, it is however not excluded that in aggregate these variants may eventually contribute to improved risk stratification [8].

## Rare Monogenic Causes of Stroke

Monogenic (Mendelian) disorders are responsible for a very small proportion of strokes, probably less than 1 % (this proportion being larger among younger stroke patients) [9]. The mechanisms by which these monogenic disorders result in stroke vary substantially. The main monogenic disorders causing stroke are presented in Table 1. Only monogenic diseases for which stroke is one of the main clinical manifestations are presented, and we have not included inherited cardiopathies, such as familial atrial fibrillation, or vascular malformations, such as familial cavernomas.

**Table 1:** Monogenic causes of ischemic stroke

Disease	Gene	Inheritance mode	Other clinical features (main)
Monogenic diseases causing small artery occlusion (lacunar) ischemic stroke			
CADASIL [78]	*NOTCH3*	autosomal dominant	migraine with aura, mood disturbance, progressive cognitive impairment
CARASIL [79, 80]	*HTRA1*	autosomal recessive	alopecia, dementia, gait disturbance, low back pain, mood disturbance
RVCL [81–84]	*TREX1*	autosomal dominant	visual loss, stroke, dementia, seizures, headache, personality disorders
Monogenic diseases causing large artery and small artery occlusion (lacunar) ischemic stroke			
Sickle cell disease [85, 86]	*HBB*	autosomal recessive	more seldom hemorrhagic stroke, vaso-occlusive or painful crisis, retinopathy, chronic leg ulcers, increased susceptibility to infection, anemia
Homocystinuria [87, 88]	CBS*	autosomal recessive*	thromboembolism, mental retardation, ectopia lentis, skeletal abnormalities
Fabry disease [89–91]	*GLA*	X-linked	acroparesthesia, hypohidrosis, angiokeratoma, chronic kidney disease, cardiomyopathy
PXE [92–94]	*ABCC6*	autosomal recessive	peau d’orange lesions, ocular complications, hypertension, peripheral artery disease, coronary artery disease, gastrointestinal bleeding
Monogenic diseases causing ischemic stroke of other etiologies			
Vascular EDS [95–98]	*COL3A1*	autosomal dominant	dissection and rupture of arteries, rupture or hollow organs, easy bruising, thin skin with visible veins
Marfan syndrome [99–102]	FBN1*	autosomal dominant	aortic root dilatation and dissection, ectopia lentis, marfanoid habitus, dural ectasia, pneumothorax
Monogenic mitochondrial disorders			
MELAS [103–105]	*tRNA Leu*	maternal inheritance	seizures, recurrent headaches, anorexia, recurrent vomiting, myopathy with exercise intolerance
Monogenic diseases causing intracerebral hemorrhage			
CAA [106–110]	*APP, CST3*	autosomal dominant	dementia, transient neurological symptoms and seizures
COL4A1 syndrome [111–115]	*COL4A1*	autosomal dominant	more seldom lacunar ischemic stroke, porencephaly, intracranial aneurysms, retinal arteriolar tortuosities and haemorrhage, cataract, Axenfeld-Rieger syndrome, hematuria, renal cysts, mild renal failure, muscle cramps

*Most frequent form

*CAA* cerebral amyloid angiopathy, *CADASIL* cerebral autosomal dominant arteriopathy with subcortical infarcts and leukoencephalopathy, *CARASIL* cerebral autosomal recessive arteriopathy with subcortical infarcts and leukoencephalopathy, *COL4A1* collagen 4A1, *EDS* Ehlers-Danlos syndrome, *MELAS* mitochondrial myopathy, encephalopathy, lactic acidosis and stroke-like episodes, *PXE* pseudoxanthoma Elasticum, *RVCL* retinal vasculopathy and cerebral leukodystrophy

As has been shown for other diseases [8], it has been hypothesized that some genes harboring causal mutations for monogenic forms of stroke may also contain common genetic polymorphisms associated with complex stroke risk. There is some preliminary indication of that for the *COL4A2* gene, for instance. Indeed, common variants in this gene, which harbors mutations for a Mendelian disease causing intracerebral hemorrhage (Table 1), were found to be associated with an increased risk of multifactorial deep intracerebral hemorrhage [10, 11].

## Role of Genetic Risk Factors in Common Multifactorial Stroke

There is evidence from twin studies [12], and from studies on the family history of stroke [13–15], that genetic factors substantially contribute to stroke susceptibility. However, there is important heterogeneity of heritability estimates. Many studies combined ischemic and hemorrhagic stroke, and only a few studies considered ischemic stroke subtypes [16]. Recently, with the advent of genome-wide genotyping (which consists of genotyping several hundreds of thousands or millions of genetic variants distributed across the genome), novel approaches have been developed that enable estimation of the heritability of diseases in the absence of familial information, based solely on genome-wide genotypes [17]. This “pseudo-heritability” corresponds to the proportion of phenotypic variance explained by genome-wide genotypes [18]. Recently, the pseudo-heritability of stroke has been estimated based on data from large genome-wide association studies, confirming a substantial heritability [19, 20], but also important differences according to stroke subtypes. Pseudo-heritability estimates were 40.3 % for large artery ischemic stroke, 32.6 % for cardioembolic ischemic stroke, 16.1 % for small artery occlusion ischemic stroke, 73 % for lobar intracerebral hemorrhage, and 34 % for deep intracerebral hemorrhage. Heritability estimates may increase if phenotyping is more accurate, as was for instance shown for small artery occlusion ischemic stroke, the heritability of which reached 24 % when considering only patients with multiple lacunar infarcts or associated extensive white matter hyperintensities, reflecting underlying cerebral small artery disease [21].

The underlying genetic model is believed to be multifactorial, with numerous genetic polymorphisms that each confers a small increase in risk, and several environmental risk factors also of small effect size, and possible interaction of these risk factors with each other [22, 23]. The most commonly studied type of genetic variation underlying the risk of complex diseases like stroke is single nucleotide polymorphisms (SNPs), of which several million have been identified across the genome.

Given the small effect size of genetic risk variants expected for stroke, large numbers of individuals are required to reach sufficient statistical power, in the range of several thousand at least. Hence, identifying such variants requires large collaborative efforts, made possible through the creation of international consortia, such as the International Stroke Genetics Consortium (ISGC, www.strokegenetics.org), the NINDS Stroke Genetics Network, the Cohorts of Heart and Aging Research in Genomic Epidemiology (CHARGE) consortium, and the METASTROKE consortium [24–26].

**Fig. 2:**
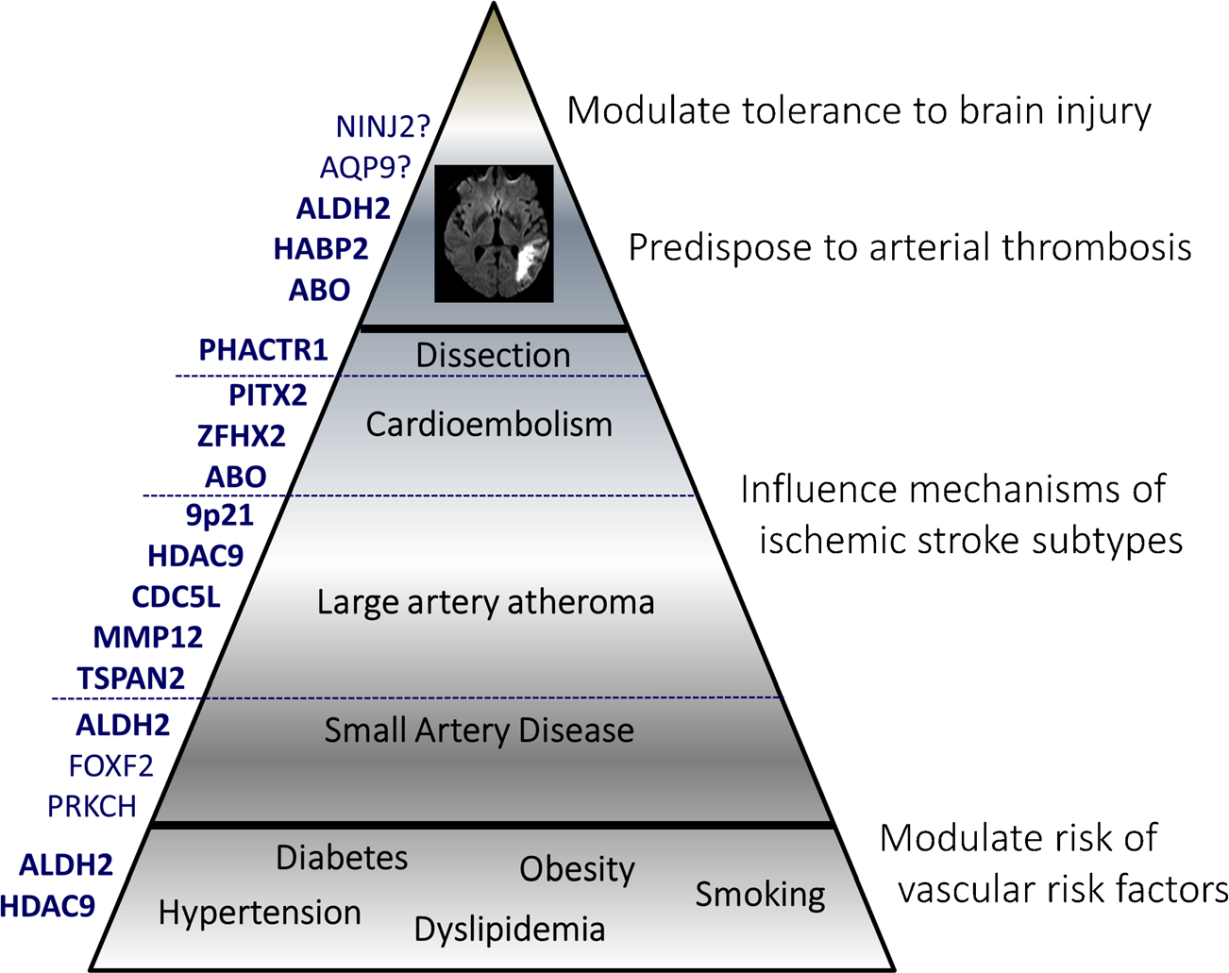
Genetic risk factors for stroke can act at various levels, example for ischemic stroke. *Left side* Risk loci identified to be associated with ischemic stroke. *Right side* Theoretical mechanisms by which genetic factors may modulate ischemic stroke risk

Genetic variants predisposing to stroke could act at various levels (Fig. 2), e.g., by increasing the risk of and susceptibility to “conventional” stroke risk factors such as hypertension or diabetes, by influencing specific mechanisms underlying stroke, such as atheroma or atrial fibrillation, by alterating coagulation pathways and predisposing to arterial thrombosis or bleeding, or by modifying tolerance to brain ischemia and more largely brain injury [27].

Recently, large collaborative efforts have identified a number of common genetic risk variants associated with an increased risk of stroke, both ischemic and hemorrhagic stroke [28•, 29–34]. Earlier studies had consisted of testing the association of stroke with a few genetic variants in one or few candidate genes, selected based on a priori hypotheses on the mechanisms underlying the disease, leading to disappointing results, as most associations that were identified could not be confirmed in independent samples [35]. Most robust genetic associations with stroke were identified through genome-wide association studies, an agnostic approach that consists of genotyping a very large number of genetic variants (“markers”) across the genome and test their association with a phenotype, without any a priori hypothesis on the underlying biology [22, 36, 37]. This approach has led to the identification of a very large number of genetic associations with various traits and diseases that have been convincingly replicated in independent samples, interestingly mostly near previously unsuspected genes, thus providing new hypotheses on the underlying biology [8].

In the early phase of genome-wide association studies, most genome-wide significant associations for stroke were identified for specific ischemic or hemorrhagic subtypes [28•, 29–31, 38]. However, analysis of shared genetic variation across stroke subtypes has also shown high genetic correlation between some stroke subtypes, such as the large-artery atherosclerosis and small artery disease subtypes of ischemic stroke [24]. Moreover, with increasing sample size through large collaborative efforts, more recently multiple studies have also reported loci associated with all ischemic stroke and all stroke [33•, 34, 39•, 40•, 41•, 42•]. Genetic risk variants associated with all stroke are expected to be either acting at the upstream end of the pathophysiological cascade, by modulating the risk of medical conditions increasing the risk of all types of stroke (such as hypertension) or at the downstream end by modulating the brain’s susceptibility to ischemic injury (e.g., via increased risk of thrombosis) or the brain’s tolerance to brain injury at large (e.g., through affecting neuroprotective pathways, Fig. 2). However, particularly large numbers are likely required to reveal such genetic risk loci for stroke, given their small expected effect size in light of the numerous concomitant risk factors required for this highly heterogeneous condition to occur.

### Genetic Risk Factors for Ischemic Stroke

Genome-wide significant associations with ischemic stroke are summarized in Table 2. A *p* value <5 × 10^−8^ is required to declare genome-wide significance, to account for approximately one million independent statistical tests performed genome-wide.

**Table 2:** Genome-wide risk loci for complex forms of ischemic and hemorrhagic stroke

Locus	Lead-SNP	Chromosome	Position	Phenotype	Risk Allele	Risk Allele Frequency	N^a^	OR	P
Ischemic stroke									
*TSPAN2* [51•]	rs12122341	1	115655690	LAS	G	0.25	20,941/364,736	1.19	1.30 × 10^−9^
*PITX2* [29]	rs6843082	4	111718067	CE	G	0.21	2365/12,389	1.36	7.8 × 10^−16^
*FOXF2* [39•]	rs12204590	6	1337393	AS, *SVD* ^*‡*^	A	0.21	24,164/155,765	1.08	1.48 × 10^−8^
*CDC5L* [32]	rs556621	6	44594159	LAS	A	0.33	400/1172	1.62	3.9 × 10^−8^
*HDAC9* [29]	rs2107595	7	19049388	LAS	A	0.16	2167/12,389	1.39	2.0 × 10^−16^
*ABO* [40•]	rs505922	9	136149229	LAS, IS	A	0.19	26,127/53,788	1.09	4.3 × 10^−8^
*HABP2* [41•]	rs11196288	10	115057443	IS	G	0.05	5508/29,713	1.41	9.5 × 10^−9^
*MMP12* [28•]	rs660599	11	102729757	LAS	A	0.19	3197/62,912	1.18	2.6 × 10^−8^
*NINJ2* [34]	rs11833579	12	775199	IS	A	0.23	1164/18,058	1.41	2.3 × 10^−10^
*SH2B3/ALDH2* [33•]	rs10744777	12	112233018	IS, SVD	T	0.66	17970/70,764	1.1	7.1 × 10^−11^
*PRKCH* [59]	rs2230500	14	61924239	SVD	A	0.19	2246/2971	1.4	5.1 × 10^−7^
*AQP9* [42•]	rs4471613	15	58551694	AS	A	0.02	1592/13,153	2.27	3.9 × 10^−8^
*ZFHX3* [29]	rs879324	16	73068678	CE	A	0.19	2365/12,389	1.25	2.3 × 10^−8^
Intracerebral hemorrhage									
*PMF1* [30•]	rs2984613	1	156197380	ICH (deep)	C	0.32	881/1481	1.33	2.2 × 10^−10^
*APOE* [38]	rs429358	19	45411941	ICH (lobar)	ε2	0.07	931/3744	1.82	6.6 × 10^−10^
*APOE* [38]	rs429358	19	45411941	ICH (lobar, *deep* ^b^)	ε4	0.12	931/3744	2.2	2.4 × 10^−11^

^a^N cases / N controls

^b^Not genome-wide significant for the subtypes in *italic*

*AS* all stroke, *IS* ischemic stroke, *CE* cardioembolic, *LAA* large artery atherosclerosis, *ICH* intracerebral hemorrhage

Genetic loci found to be associated with cardio-embolic ischemic stroke were already known risk loci for atrial fibrillation (*PITX2* and *ZFHX3*), consistent with the fact that atrial fibrillation is by far the most common source of cardioembolic events [43–45]. Recent data suggest that the *PITX2* locus could perhaps also contribute to stroke risk independent of atrial fibrillation [46]. Indeed, *Pitx2−/−* mutant mice were shown to exhibit reduced and discontinuous smooth muscle actin staining of cerebral vessels and increased cerebral vessel density. Moreover, *PITX2* variants were found to be associated with increasing white matter hyperintensity burden on brain MRI in large population-based samples [46]. Evidence for association of the *PITX2* locus with small artery occlusion ischemic stroke, however, is lacking to date [29].

In contrast with cardio-embolic IS, genetic variants found to be associated with large artery IS at the genome-wide level (*HDAC9*, *MMP12*, *CDC5L*, and *TSPAN2*) are all in loci previously unsuspected at the time of discovery [28•, 29, 32]. The *HDAC9* locus was subsequently also identified as a risk locus for coronary artery disease [47] and pulse pressure [48]. Interestingly, risk allele carriers of the lead *HDAC9* susceptibility variant for large artery ischemic stroke (rs2107595) were found to be associated with increased mRNA levels of *HDAC9* [49]. Moreover, compared with *Hdac*9+/+*Apoe*−/− mice, *Hdac*9−/−*Apoe*−/− mice exhibited reduced atherosclerotic lesion size throughout the aorta [49], making HDAC9 a plausible target for pharmacologic prevention of atherosclerosis. The *MMP12* locus was identified by implementing an age-at-onset informed genome-wide association analysis (i.e., a regression analysis conditioning on age-at-onset) and was significantly overexpressed in carotid plaques [28•]. The age-at-onset analysis was driven by the assumption that early onset stroke may have increased genetic liability [50]. Most recently, a large collaborative study conducted by the NINDS-SiGN consortium identified common variants near *TSPAN2* to be associated with large artery ischemic stroke [51•]. Other variants near *TSPAN2* have been implicated in migraine, but they are not in linkage disequilibrium with (i.e., not correlated with) the stroke risk variants. *TSPAN2* has also been implicated in neuro-inflammation [52]. In addition to these genome-wide significant findings, a few candidate gene-based associations (requiring a less stringent threshold for significance) have been robustly replicated in large independent studies, such as the chr9p21 locus (rs2383207) or the *ABO* locus (rs505922) on chromosome 9 with large artery ischemic stroke [29, 53, 54]. The *ABO* locus also showed association with cardioembolic ischemic stroke and has recently also been shown to reach genome-wide significance with all ischemic stroke [40•, 54]. Both the chr9p21 and the *ABO* locus were independently found to be associated with coronary artery disease, and *ABO* is also a risk locus for venous thromboembolism [47, 55].

Until recently, efforts at identifying genetic risk variants for small artery occlusion ischemic stroke have yielded little, with no genome-wide significant finding in the largest published GWAS, despite a similar sample size compared to the aforementioned ischemic stroke subtypes [29]. As pseudo-heritability estimates were also smaller for this subtype (16 vs. 40 % for large artery and 33 % for cardioembolic ischemic stroke) [19], it has been hypothesized that this could reflect a lesser contribution of genetic factors to this ischemic stroke subtype. Another possible explanation is the heterogeneity and imprecision in the phenotype definition of small artery occlusion ischemic stroke, when following the most commonly used Trial of Org 10172 in Acute Stroke Treatment (TOAST) stroke subtyping algorithm [56]. Genetic liability to small artery occlusion ischemic stroke may also differ according to ethnic background, as this stroke subtype is much more prevalent in Asian populations. A significant association of a variant in the *PRKCH* gene with small artery occlusion ischemic stroke was described in Japanese and Chinese populations. This variant is monomorphic in European populations and no association was found either with nearby variants in *PRKCH* and small artery occlusion ischemic stroke in Europeans [29, 57–59]. Interestingly, a recent large genome-wide association study of incident stroke conducted by the CHARGE consortium, followed by replication in studies with prevalent stroke (mostly from the NINDS-SiGN and METASTROKE consortia), identified common variants on chr6p25 near *FOXF2* to be associated with all stroke, and this association was particularly strong with small artery occlusion ischemic stroke compared to other stroke subtypes [39•]. The same variants near *FOXF2* were also found to be associated with larger white matter hyperintensity burden in older stroke-free community persons. Interestingly, patients with a rare monogenic ophthalmologic condition due to segmental deletions encompassing *FOXF2* (Axenfeld-Rieger syndrome) also exhibited extensive white matter hyperintensities. Conditional deletion of *Foxf2* in adult mice led to cerebral infarction, reactive gliosis, and microhemorrhage. In zebrafish, foxf2b−/− mutants showed decreased smooth-muscle cell and pericyte coverage, suggestion that *FOXF2* may be involved in mural cell differentiation [39•].

In addition to *FOXF2*, other loci have also recently been reported to be associated with all ischemic stroke or all stroke at a genome-wide significant level. The first to be reported was an association with the chr12p13 locus, near *NINJ2*, associated with incident stroke and ischemic stroke in particular in prospective population-based cohort studies participating in the CHARGE consortium [34]. However, although the same association was reported in several Asian studies [60], it could not be replicated in a large hospital-based genetic association study of prevalent stroke in European populations [61]. One potential explanation for these discrepant results, beside type I error (false positive finding), is that the chr12p13 locus could be associated with stroke severity and mortality more than with stroke risk. Indeed, in hospital-based cross-sectional studies, given high early mortality rates of stroke, death might occur very early before hospitalization or before samples can be taken. Conversely, in prospective cohort studies, severe strokes leading to early death are included, as blood samples were taken at recruitment in the study, before stroke onset. Allelic heterogeneity at this locus, caused by multiple rare, low frequency, and common variants with disparate effects on risk, may also explain the difficulties in replicating the original GWAS results [62]. The second genetic risk locus for all ischemic stroke (chr12q24.12) was identified in a case–control dataset with over 17,000 ischemic stroke patients and found to be equally associated with all subtypes of ischemic stroke. The single nucleotide polymorphism (SNP) showing the most significant association is in linkage disequilibrium with a non-synonymous variant in *SH2B3* and is associated with gene expression of *ALDH2*, pointing to a potential role of these two genes in the association [33•]. The same locus was previously associated with blood pressure and coronary artery disease [47, 63, 64]. Two other recent studies have also described associations of variants near *HABP2* and *AQP9* to be associated with all ischemic stroke and all stroke, respectively [40•, 41•]. *HABP2*, which encodes for an extracellular serine protease involved in coagulation, fibrinolysis, and inflammation, was identified through a young-onset stroke GWAS where only stroke cases with age <60 years were studied, and replicated in an independent dataset [40•]. *AQP9* was identified through the first large-scale GWAS on individuals of African ancestry [41•]. Though *AQP9* reached genome-wide significance in the discovery stage, it did not show evidence of replication in individuals of European ancestry and still needs to be confirmed in independent large studies in individuals of African ancestry.

Novel genetic risk loci have also recently been discovered for other less common but well characterized ischemic stroke etiologies, such as cervical artery dissections, a major cause of ischemic stroke in young adults [65•]. This large collaborative study from the CADISP consortium found the minor allele of a common variant at *PHACTR1* to be associated with a lower risk of cervical artery dissection. Interestingly, the same allele was independently found to be associated with a lower risk of migraine (especially without aura) and with an increased risk of coronary artery disease [47, 52, 65•], suggesting that this locus may play a pivotal role in vascular biology. The cervical artery dissection risk allele is associated with increased expression of *PHACTR1* in certain tissues [66]. The function of *PHACTR1* is poorly understood. Experimental studies revealed a pivotal role in vascular tube formation and actin polymerization [40•, 41•] Upregulation of *PHACTR1* by TGFβ has been described [42•], potentially pointing to a connection with the TGFβ signaling pathway. A role in mechanotransduction has also been suggested [67].

### Genetic Risk Factors for Intracerebral Hemorrhage

A highly significant and robust association with intracerebral hemorrhage was demonstrated for the *APOE* locus in a large candidate gene association study on 2189 cases and 4041 controls [38]. Both *APOEε2* and the *APOEε4* alleles were associated with lobar intracerebral hemorrhage at a “genome-wide significance level”, with odds ratios of 1.82 (*p* = 6.6 × 10^−10^) and 2.20 (*p* = 2.4 × 10^−11^), respectively. Associations were even stronger when restricting the analysis to patients with definite or probable underlying cerebral amyloid angiopathy (CAA). *APOEε4* was also associated with an increased risk for deep intracerebral hemorrhage, a location not affected by CAA, at a lower significance level (odds ratio at 1.21, *p* = 2.6 × 10^−4^), suggesting that mechanisms linking *APOEε4* to intracerebral hemorrhage may expand beyond CAA-mediated effects [38, 68]. In a subsequent analysis, the authors also demonstrated a strong association of the *APOE* locus with hemorrhage size and growth [69].

The International Stroke Genetics Consortium recently published the first genome-wide association analysis of intracerebral hemorrhage, based on 1545 patients with intracerebral hemorrhage (664 lobar and 881 non-lobar) and 1481 controls. This study identified one novel genome-wide significant locus on chromosome 1q22 associated specifically with an increased risk of non-lobar (deep) intracerebral hemorrhage, with replication in an independent sample [30•]. Interestingly, this locus was also recently found to be associated at a genome-wide level with increasing white matter hyperintensity burden, the most plausible pathophysiological link between both associations being an increased liability to cerebral small artery disease [70•, 71]. In a recent population-based GWAS of incident stroke, the same locus also showed highly suggestive, although not genome-wide significant, association with incident ischemic stroke [39•]. The identified genetic risk variants are located in a region that contains *PMF1* and *SLC25A44*, and they are associated with expression levels of a nearby gene, *SEMA4A* [30•]. The results of this study also emphasize the biological heterogeneity across ICH subtypes, as this association was found exclusively for non-lobar ICH.

## Discussion

In summary, in the past few years, the advent of high-throughput genotyping technologies and the creation of large international consortia have brought important new insight into complex stroke genetics. Twelve new risk loci for ischemic stroke have been discovered, although two of these have either not been confirmed or were subject to controversy, with inconsistent replication. Five of these loci showed association with all ischemic stroke and all stroke; four, with large artery ischemic stroke; and two, with cardioembolic ischemic stroke. One all-stroke risk locus showed predominant association with small artery occlusion ischemic stroke, but did not reach genome-wide significance in this subtype. Two new loci were identified for intracerebral hemorrhage.

These discoveries of common genetic variants associated with stroke and its subtypes have substantially broadened our knowledge of the underlying pathophysiology. Recent findings have also emphasized the need to carefully consider ischemic and hemorrhagic stroke not as single entities, but as composite entities comprised of various underlying diseases, some of which may share common mechanisms. Our understanding of stroke genetics may be enriched by exploring MRI-based endophenotypes for specific stroke subtypes, such as white matter hyperintensity burden, a marker of cerebral small artery disease, which is strongly correlated with small artery occlusion ischemic stroke. Recently, five genome-wide significant risk loci for white matter hyperintensity burden have been identified (chr17q25, chr10q24, chr2p21, chr1q22, and chr2p16) [70•].

So far, most studies of complex stroke genetics have focused on common single-nucleotide polymorphisms, and, as for other complex diseases, these identified common risk variants explain only a small proportion of the disease heritability [72]. Other types of variation, such as low frequency (1–5 %) or rare (<1 %) single nucleotide variants, or structural variation such as copy number variants, have been insufficiently explored. New genome-wide genotyping arrays now also partly cover lower frequency variants, and more importantly, the advent of next generation sequencing technologies has made sequencing of large samples more accessible and thus opened new avenues for studying unexplored rare variants as well as structural variation [73, 74]. So far, no major results have emerged yet for common stroke, but there are encouraging preliminary findings.

One important discovery of genome-wide association studies is the amount of pleiotropy or shared genetic variation between stroke and other complex phenotypes, which also tells us more about disease mechanisms [75]. Substantial overlap was, for instance, observed between the genetic risk of ischemic stroke, and particularly the large artery subtype, with coronary artery disease, thus contributing to a better understanding of common underlying biological pathways [76]. Pleiotropy may be particularly helpful in understanding the biological mechanisms underlying statistical association, when specific risk loci are shared across phenotypes, such as the *PHACTR1* gene that is assumed to play a central role in vascular biology.

As part of the International Stroke Genetics Consortium, the METASTROKE collaboration, the NIH-funded SiGN initiative, and the CHARGE consortium, efforts are ongoing to perform genome-wide association studies of stroke in much larger samples of patients, in order to increase power to detect genetic association with stroke and its subtypes. These projects take advantage of the most recent 1000 genomes reference panel (www.1000genomes.org) that enables more reliable imputation (statistical inference) of genotypes for millions of genetic variants that have not been genotyped. Availability of newer reference panels like the Haplotype Reference Consortium panel (http://www.haplotype-reference-consortium.org) that enable imputation of low frequency variants also provide an exciting opportunity. Efforts are now being made to expand genetic studies to non-European ethnic groups, an essential step to enhance the discovery of stroke risk loci, as was shown for other conditions [77]. In parallel data on rare variants obtained through exome chip genotyping, whole exome and whole genome sequencing is being accrued. In addition to uncovering new risk loci, sequencing will likely help fine-map genetic risk loci discovered by genome-wide association studies and facilitate the identification of the underlying causal variant and gene. Our understanding of the genetic underpinnings of stroke will be further enriched by combining genomic information with trancriptomic, epigenomic, proteomic, metabolomic, and other -omic data. These can provide crucial information to identify the causal variant and gene underlying the observed statistical associations, an essential step for the design of experimental studies aiming at deciphering the underlying biology.
